# A Narrative Review of the W, X, Y, E, and NG of Meningococcal Disease: Emerging Capsular Groups, Pathotypes, and Global Control

**DOI:** 10.3390/microorganisms9030519

**Published:** 2021-03-03

**Authors:** Yih-Ling Tzeng, David S. Stephens

**Affiliations:** 1 Division of Infectious Diseases, Department of Medicine, Emory University School of Medicine, Atlanta, GA 30322, USA; ytzeng@emory.edu; 2 Department of Microbiology and Immunology, Emory University School of Medicine, Atlanta, GA 30322, USA

**Keywords:** *Neisseria meningitidis*, capsule, meningococcal group, nongroupable, meningococcal carriage, invasive meningococcal disease, meningococcal urethritis

## Abstract

*Neisseria meningitidis*, carried in the human nasopharynx asymptomatically by ~10% of the population, remains a leading cause of meningitis and rapidly fatal sepsis, usually in otherwise healthy individuals. The epidemiology of invasive meningococcal disease (IMD) varies substantially by geography and over time and is now influenced by meningococcal vaccines and in 2020–2021 by COVID-19 pandemic containment measures. While 12 capsular groups, defined by capsular polysaccharide structures, can be expressed by *N. meningitidis*, groups A, B, and C historically caused most IMD. However, the use of mono-, bi-, and quadrivalent-polysaccharide-conjugate vaccines, the introduction of protein-based vaccines for group B, natural disease fluctuations, new drugs (e.g., eculizumab) that increase meningococcal susceptibility, changing transmission dynamics and meningococcal evolution are impacting the incidence of the capsular groups causing IMD. While the ability to spread and cause illness vary considerably, capsular groups W, X, and Y now cause significant IMD. In addition, group E and nongroupable meningococci have appeared as a cause of invasive disease, and a nongroupable *N. meningitidis* pathotype of the hypervirulent clonal complex 11 is causing sexually transmitted urethritis cases and outbreaks. Carriage and IMD of the previously “minor” *N. meningitidis* are reviewed and the need for polyvalent meningococcal vaccines emphasized.

## 1. Introduction

*Neisseria meningitidis* (the meningococcus), a Gram-negative pathogen of humans, causes epidemic meningitis and rapidly fatal sepsis in many parts of the world. *N. meningitidis* is usually a commensal inhabitant of the human respiratory tract, is isolated from the nasopharynx of 3–20% of healthy individuals in the absence of outbreaks or crowding [1,2] and is transmitted from person to person by close contact of large aerosolized droplets or with oral or nasal secretions. Recent studies have suggested a global decline in overall meningococcal carriage.

For both children and adults, carriage of *N. meningitidis* can be an immunizing event, resulting in systemic protective immune response. While in most instances, the acquisition of meningococci in the upper respiratory tract does not result in invasive disease, invasive meningococcal disease (IMD), even with antibiotic therapy and supportive care, has a mortality rate that remains at 10–15%. Factors determining the establishment of carriage versus the development of invasive meningococcal disease following acquisition include expression of capsule and other bacterial virulence determinants (reflected in virulent genotypes, such as clonal complex (cc) 5 and cc7 (group A), cc41/44, cc32, cc18, cc269, cc8, and cc35 (group B), and cc11 (group C)) and host susceptibility.

The meningococcus often produces a capsular polysaccharide (CPS) in which structural differences form the basis of the historic serogroup typing system. Although there are 12 capsular groups expressed by *N. meningitidis* and defined genetically [3], three groups, A, B, and C, have been associated with significant invasive disease. Group A (MenA) *N. meningitidis* expressing a homopolymeric (α1 → 6) *N*-acetylmannosamine 1-phosphate capsule caused large pandemic outbreaks globally through much of the 20th century that persisted especially in the meningitis belt of sub-Saharan Africa until introduction of the MenA conjugate vaccine, MenAfriVac, in 2010. Meningococci of groups B and C (MenB and MenC), which are (α2 → 8)- and (α2 → 9)-linked homopolymers of sialic acid (*N*-acetylneuraminic acid), respectively, cause clusters or local outbreaks (MenC) or localized longer outbreaks and hyperendemic disease (MenB) throughout the world and have been responsible for most sporadic meningococcal disease in developed countries. The “minor” groups, X, Y, and Z were first identified by Slaterus in 1961 and groups E (29E) and W (W135) were identified in 1968. The group Y (MenY) capsular polymer is composed of alternating D-glucose and partially O-acetylated sialic acid, while the group W (MenW) capsular polysaccharide is an alternating D-galactose and partially O-acetylated sialic acid. MenY and MenW have emerged as groups causing epidemic outbreaks and global disease since 1995. Group X meningococci (MenX) expressing homopolymer of (α1→4) *N*-acetylglucosamine 1-phosphate [4] also now cause outbreaks and endemic disease in parts of sub-Saharan Africa. Meningococcal group E (MenE) expresses a capsule consisting of alternating D-galactosamine and 2-keto-3-deoxyoctulosonate (KDO) residues [5] and nongroupable (MenNG) strains, either with capsule null locus (*cnl*) or unencapsulated due to inactivation of capsule synthesis, rarely but are now identified as a cause of invasive disease especially in immunocompromised individuals. Recently an unencapsulated cc11 meningococcal pathotype causing sexually transmitted urethritis cases, outbreaks, and disease clusters has also been recognized [6,7,8].

Meningococcal disease incidence has historically been cyclical in nature [9]. Epidemiological studies of *N. meningitidis* have clearly shown that IMD varies over time, influenced by the circulating meningococcal groups and clonal complex genotypes, by geographic locations and by the host populations affected [10]. In this report, we provide an overview of carriage and invasive disease caused by “minor” *N. meningitidis* groups W, X, Y, and E as well as nongroupable meningococci causing invasive disease and sexually transmitted infections. During the COVID-19 pandemic, many countries have experienced a significant, sustained reduction in invasive diseases due to *N meningitidis* [11] and other invasive respiratory pathogens (*Hemophilus influenzae* and *Streptococcus pneumoniae*, but not *S. agalactiae*), coinciding with the introduction of COVID-19 containment measures (medRxiv preprint, https://doi.org/10.1101/2020.11.18.20225029, access on 20 November 2020).

## 2. Minor *N. meningitidis* Capsular Groups

### 2.1. Group W

Group W meningococci (MenW) prior to 2000 was an infrequent cause of meningococcal disease. The first global epidemic caused by MenW was detected in 2000, after the Hajj pilgrimage to Mecca, Saudi Arabia [12] and was attributed to a specific MenW:cc11 lineage, referred to as the Hajj lineage [13]. The MenW attack rate was high among the pilgrims and household contacts of returning pilgrims. The emergence of MenW in a background of the hypervirulent cc11 lineage was likely related to capsule switching events [14]. Subsequently, MenW strains have continued to evolve and cause global disease. The global spread of diverse cc11 lineages expressing capsular groups C, B, and W, resolved by genomic typing, divided the invasive MenW:cc11 lineage into the Hajj and the South American sublineages [14]. The South American sublineage, with the spread in Brazil, Argentina, and Chile, emerged in the mid-2000s [15] and has been further divided into the original U.K. 2009 lineage [14] and the newly emerging novel U.K. 2013 lineage [16].

Surveillance data from 13 European countries revealed an increase in MenW IMD in the period of 2013–2017 [17]. While the annual incidence of IMD remained stable during that time, the incidence and the proportion of MenW IMD among all IMD increased significantly. Average annual percentage increase in MenW incidence during this period was significant for the Netherlands (133%), Norway (86%), Spain (62%), Sweden (58%), Switzerland (44%), Germany (35%), and England (23%). The proportion of MenW among all IMD cases varied considerably between countries. The proportion of MenW was lowest in Portugal, Greece, and Poland (2–3%), while it was highest in Switzerland, the Netherlands, and England (22–24%) [17]. Of the MenW IMD isolates analyzed by multilocus sequence typing (MLST), 80% belonged to cc11 but cc22, cc174, and cc865 also caused disease. The proportion of MenW cc11 increased from 64% in the year 2013 to 86% in the year 2016. The increase in MenW IMD in England and Wales since 2009 has been mainly due to the novel U.K. 2013 lineage [16]. MenW IMD incidence, with an associated case fatality rate of 28.6%, increased from 0.02/100,000 in 2013 to 0.29/100,000 in 2017 in the Republic of Ireland and the Ireland MenW isolates clustered among both the original UK 2009 and the novel U.K. 2013 lineages [18]. Sweden had a low incidence of MenW IMD with an average incidence of 0.03 case/100,000 population from 1995 to 2014; however, the incidence of MenW increased 5-fold in 2015. This increase in MenW IMD was due to isolates belonging to the novel U.K. 2013 lineage that were introduced into Sweden in 2013 and have since been the dominant lineage of MenW [19]. The increases seen in Europe follows an increasing incidence of MenW in South America since 2004 [15], in Australia since 2013 [20], and in Canada since 2015 [21].

MenW became the predominant meningococcal capsule group in Australia in 2016 [20]. In 2017, an unprecedented outbreak of MenW infection occurred among the Indigenous pediatric population of Central Australia. Among these cases were atypical manifestations, including meningococcal pneumonia, septic arthritis, and conjunctivitis [22]. The Canadian MenW:cc11 isolates have been shown to be distinct from the traditional MenW:cc22. Both the Hajj-related and non-Hajj MenW:cc11 strains were associated with IMD in Canada [21].

A review of IMD in the Asia–Pacific region conducted by Global Meningococcal Initiative (GMI) recently reported that the predominant capsular groups were B, W, and Y in Australia, New Zealand, and China [23]. MenW circulation is significant across the Asia–Pacific region. The Philippines reported that 16.7% of sterile specimens collected in 2018 were MenW. As noted, a higher percentage (28%) of MenW was reported in Australia and a similar proportion (30%) of MenW cases was reported in New Zealand during the same period. An update on the global spread of cc11 provided during the GMI meeting highlighted the presence of the MenW:cc11 Hajj strain sublineage in Russia and Bangladesh; the MenW:cc11 South American strain sublineage in Russia, Japan, and New Zealand; the MenW:cc11 Chinese strain sublineage in China and Japan; and a further distinct MenW:cc11 strain in Bangladesh [23].

The introduction of the MenAfriVac vaccine in 2010 dramatically reduced MenA cases in 26 countries of the meningitis belt but magnified other groups as significant problems in the region, in particular groups C, W, and X. While MenW:cc11 cases have been reported in the African meningitis belt since the late 1990s and no epidemics have occurred since 2001, MenW:cc11 seems to have reemerged after 2010 [24]. In 2016, Togo experienced its second largest epidemic of bacterial meningitis since 1997, where 91.5% were due to MenW:cc11 [25]. The MenW:cc11 isolates collected in Burkina Faso during 2011–2012, Mali during 2012, and Niger during 2015 have been shown to descend from the strain identified during the Hajj-related outbreak of 2000 [26,27,28]. On the other hand, the MenW:cc11 isolates from Central African Republic in 2015–2016 grouped together in a genetic cluster separated from the Anglo-French Hajj sublineage and the South American/UK sublineage. These data appear to support a multifocal emergence of MenW:cc11 strains. The epidemiology of IMD in South Africa over 14 years [29] shows that MenW accounts for 49.5% IMD. Patients with MenW were 3 times more likely to present with severe disease than those with MenB, and HIV was associated with an increased risk of IMD, especially for MenW and MenY diseases.

### 2.2. Group X

Sporadic cases of IMD caused by *N. meningitidis* group X (MenX) have been reported in industrialized countries since 1980s [30,31,32] but since the late 1990s, MenX has emerged as a cause of IMD outbreaks in sub-Saharan African countries [33,34,35,36,37,38,39]. The PubMLST database (>75,000 isolate records, updated on 01/03/2021) contains a collection of 636 MenX isolates, 1961–2019. MenX is the latest group to cause large localized outbreaks in Kenya [36,37], Niger [34,35], Ghana [33], Mali, and Burkina Faso [38]. A study examining MenX burden and epidemiological patterns during 2006–2010 [38] showed that in Togo during 2006–2009, MenX accounted for 16% of the bacterial meningitis cases; while in Burkina Faso during 2007–2010, MenX accounted for 7% of meningitis cases, with a significant increase from 2009 to 2010 (4–35% of all confirmed cases, respectively) [38]. With the successful vaccination campaign of the MenA conjugate vaccine starting in 2010, the significance of MenX in the African Meningitis belt has become more evident. In Burkina Faso, a few months after the introduction of MenAfriVac in 2011, among the 258 confirmed meningococcal cases, only 1.6% were MenA, whereas 59% were MenX [39]. Thus, MenX, along with MenC and MenW disease in the meningitis belt, is a major driver for a new pentavalent conjugate (ACXYW) vaccine in clinical trials for sub-Saharan Africa [40]. Of note, IMD due to MenX (cc750) has also been seen in the United Kingdom (Scotland) and elsewhere in Europe. MenX has also been identified rarely in the hypervirulent cc5, cc11, and cc41/44 backgrounds.

MenX expressing genotypes (X:4) can be efficiently transmitted and colonize the nasopharynx as was seen in military recruits in the United Kingdom [41]. In a longitudinal carriage study investigating the dynamics of meningococcal carriage during an inter-epidemic period in Ghana, the disappearance of MenA was accompanied by a sharp increase in carriage of MenX, reaching 17% and coincided with an outbreak of MenX disease [33,42]. During the peak of the MenX wave, the ratio of MenX cases to carriers was found to be between 0.1 and 0.3 per 1000 cases; while the ratio of MenA during the outbreak was between 16.8 and 42.3 per 1000 cases in the respective dry seasons [42]. These studies suggest that MenX has a disease-to-carriage ratio significantly lower than MenA and that MenX have a lower invasive potential. Like other outbreak-causing meningococci, dominant virulent clones are responsible for the majority of MenX disease. Most MenX carrier and disease isolates recovered in the African meningitis belt belonged to cc181, which has been circulating in Africa since the 1970s [43].

### 2.3. Group Y

Group Y meningococci (MenY) are frequently recovered from the nasopharynx but have historically considered less invasive than groups A, B, and C [44]. However, in the mid-1990s, the rates of IMD due to MenY increased in the United States [45], and subsequently in several European countries [46,47,48,49] as well as Israel, South America, and South Africa. Clonal complexes cc23, cc167, and cc175 have been linked to the majority of MenY IMD, but MenY IMD has also been seen with cc22 (Europe), cc174 (the United Kingdom), cc92 (Europe and South America), and cc103 (Europe).

In the mid-1990s, MenY (cc23 and cc167) emerged as a major cause of significant sporadic and hyperendemic disease in the United States. The proportion of MenY IMD cases in the United States was 2% during 1989–1991 [50], increased to 10.6% in 1992, and increase to 32.6% of reported cases in 1996 [51]. Subsequently, the proportion of MenY cases decreased in the United States [45], although still causing 15% of IMD in 2018. The increase in MenY cases has not been as prominent in neighboring Canada [52]. In 1998 at the peak of MenY incidence in the United States, a carriage study of high school students from counties in the metropolitan area of Atlanta, GA, found the rate of meningococcal carriage to be 7.7% and of these isolates, 48% were MenY [53]. However, in 2006–2007, a similar carriage study in high school students found a much lower carriage rate of <3% and a lower proportion of MenY carriage [54]. Thus, like MenX, high rates of acquisition and carriage were associated with increased disease and lower MenY carriage correlated with the decrease in MenY IMD cases [45].

Meningococcal quadrivalent conjugate vaccines against groups A, C, Y, and W (MenACWY) were licensed in the United States beginning in 2005 and coverage has steadily increased among children aged 13–17 years, from 11.7% in 2006 to 86.6% in 2018. A study comparing group distribution of IMD isolates prior to (2000–2005) and post vaccine introduction (2006–2010) reported that among all age groups, the overall IMD incidence declined over time, but there was no evidence of vaccine-induced capsular group replacement. While the incidence of IMD significantly declined in the United States, the proportion of MenY varied from 33% in 2000–2005 and 37% in 2006–2010 to 27% in 2011–2015 [55,56]. Changes in group and clonal complex were observed in isolates of both vaccine targeted and non-targeted groups. These changing profiles are likely representative of natural variation and fluctuations within meningococcal population structure. As noted, clonal complexes cc23 and cc167 accounted for most of MenY disease in both the United States and Canada [55,57,58], again suggesting that closely related strains circulate at high frequencies in a community causing sporadic disease.

MenY disease has recently emerged in Latin American countries and is characterized by clear differences from country to country [59]. Molecular characterization of MenY IMD isolates during 2000–2006 showed variable trends among 5 countries. While no increase in the frequency of MenY isolates was observed in either Brazil or Chile, the proportion of MenY IMD isolates increased in Argentina from 2002, to a level similar to those of groups C and W by 2006. In Colombia, MenY IMD isolates increased from 4% in 2000 to 50% in 2006 [60]. Venezuela also reported an increase in the proportion of cases due to MenY in 2006, representing 50% of all cases identified [59]. Again, most of the IMD isolates belonged to cc23 and cc167 [61]. Recently, IMD cases caused by penicillin- and ciprofloxacin-resistant cc23 MenY were found in El Salvador. These isolates contained a β-lactamase gene (*blaROB-1*) and a mutated DNA gyrase gene (*gyrA*) [62].

Until the last decade, MenY cases were rare in Europe, accounting for <2% of cases [63]. An emergence of MenY IMD cases was noted in several European countries after 2010. For example, in France, MenY accounted for 3% of IMD cases in 2000 to 2005, but increased to 10% in 2013 [64,65]. In Scotland, MenY IMD cases increase from 2.3% in 2010 to 17% in 2013 [65]. Further, significant increases in the incidence and the relative proportion of MenY IMD cases were found in Scandinavian countries: in Norway, the 4-year trend between 2010 and 2013 for Norway is 31–55–25–26% and in Finland, it was 38–21–24–40% [48,65]. Sweden had the highest relative proportion of MenY IMD in Europe—39% in 2010 and ~50% in the following 3 years [65]. The significantly increased MenY IMD in Sweden is mainly due to the emergence of specific cc23 clusters [47,48]. Whole-genome sequencing data of invasive MenY isolates from 1995 to 2012 in Sweden found at least three related but distinct cc23 clusters causing disease in Sweden. Thus, the increase in MenY IMD cases was not caused by the expansion of a single virulent variant [47], but was linked to increased virulence, host adaptive immunity, and transmission dynamics. Comparison to a collection of MenY isolates from England, Wales, and Northern Ireland during 2010 to 2012, which had relatively low MenY incidence, and MenY from the United States showed that the MenY cc23 clusters have a distribution spanning North America and Europe, including Sweden, over a number of years [47] but different strain types were prevalent in each geographic region.

Several carriage studies conducted in the United Kingdom have detected changes in MenY carriage in young adults over the last three decades [66,67,68,69]. MenY constituted approximately 8% of recovered isolates when carriage was assessed during 1997–1998 in first-year university students at Nottingham University, the United Kingdom [66]. During 1999–2001 in >48,000 samplings of 15–17 years old throughout the United Kingdom, MenY strains accounted for ~10% of the carriage isolates [70]. A later 2008 carriage study of first-year students carried out again at Nottingham University, the United Kingdom, found that MenY carriage reached 26% [68]. Core genome analysis of carriage-associated MenY isolates recovered in the United Kingdom during 1997–2010 reveals extensive genetic similarities to disease-associated MenY recovered during 2010–2011 [5]. Again, the majority of these MenY belong to cc23 (58% in carriage and 79% in disease) and a long-term temporal stability of MenY clones was suggested [5]. However, in South Africa, a different clonal complex was responsible for increases in MenY disease. MenY cc175 caused significant IMD and was dominant in South Africa in the early 2000s [58]. MenY is also expressed in cc11, cc32, and cc41/44 clonal complex backgrounds that are more frequently associated with other capsular groups. Interestingly, comparison of IMD cases during a 2-month lockdown period in 2020 and the same periods of 2018 and 2019 in France found significant decrease in all IMD cases from prior years, and seemed to mainly involve IMD cases due to groups B and C and W, but not IMD due to group Y and other groups or nongroupable isolates [11]. The MenY genotypes had not changed in 2020 and were cc23 [11]. The observed IMD decreased mainly in the highly transmissible and hyperinvasive isolates belonging to cc11.

### 2.4. Group E

IMD due to meningococcal group E (MenE), previously known as 29E and Z’ and first identified in 1968 [71], is infrequent and has been most often associated with immunocompromised patients [71,72]. Query of the PubMLST database shows 1003 MenE isolates in the collection. The vast majority (>75%) were from pharyngeal carriers with cc60 (42%) and cc1157 (29%) dominating. The earliest invasive MenE recorded in PubMLST is in 2000, and are predominantly cc60 (33%), cc1157 (26%), cc254 (8%), and cc178. A recent study reported the molecular characterization of three MenE IMD cases in Queensland, Australia; the emergence of these cases was attributed to a circulating cc1157 clone [73]. Globally as noted, MenE carriage is not uncommon. Historic carriage studies of first-year college students in the United Kingdom in 1997 [66] and young adults in the Czech Republic during 1993 [74] found ~6% and ~5% MenE in the respective isolate collections. A 2008 carriage study of first-year students at Nottingham University, the United Kingdom, found MenE clones highly prevalent (21–32%) in residential halls, indicative of rapid clonal expansion [67]. However, a recent carriage study performed in Australia in 2017 identified a single individual with MenE carriage from 421 first-year university students (0.2%) [75]. In contrast, an ongoing study of meningococcal carriage in participants in an STI clinic, MenE was identified in ~13% (Tzeng et al. unpublished data) of carriage isolates. While no group-specific vaccine is currently available for MenE, the protein-based group B vaccine, MenB-4C (Bexsero), contains outer membrane vesicles with multiple surface antigens that can provide cross-reactive protection. Similar data are available for MenB-FHbp (Trumenba) where bactericidal responses to groups C, W, Y, and X expressing different fHbp peptides have been shown.

### 2.5. Nongroupable

The meningococcal nongroupable (MenNG) phenotype is a result of elimination or minimal capsule production. Responsible mechanisms include down-regulation of capsule gene expression, phase variation in the capsule synthesis genes, transient or permanent inactivation of genes by insertion element movement into the capsule gene cluster (*cps*), frame-shift point mutations within an otherwise intact biosynthesis genes, or transformation/recombination events resulting in major deletion of the *cps* locus [76,77,78]. Meningococci with a capsule null locus (*cnl*), similar to *N. gonorrhoeae*/*N. lactamica*-like genetic configuration at the *cps* locus, were first identified in healthy carriers in Germany in 2000 and constituted ~16% of all recovered isolates [78]. Subsequently, additional carriage studies showed that nongroupable *cnl* meningococci are prevalent in carriage [79,80].

MenNG rarely cause invasive disease; however, *cnl* isolates have been described as a cause of IMD in immunocompetent individuals [81,82,83,84,85]. Most invasive *cnl* meningococci belong to cc198 [81,82,83] and cc192 [84,85], with cc192 being most commonly identified in Africa, but rarely elsewhere in the world [79]. Two cc198 invasive *cnl* isolates from Canada were examined using murine intraperitoneal infection model. Although no mortality was seen upon infection with the non-encapsulated MC58 derivative, 18% succumbed to infection with one *cnl* strain and 50% died after infection with the other *cnl* strain. Thus, although virulence potentials of both *cnl* strains were below that of encapsulated strain MC58, both strains exhibit a virulence phenotype [83].

While case reports in the literature across several decades have indicated that *N. meningitidis* is capable of colonizing the urogenital tract and causing sporadic cases of urethritis, cervicitis, or proctitis, very low overall incidence has been reported [86,87,88,89]. In one study of 23 meningococci isolated from the urogenital tract and rectum, two are *cnl* isolates [90]. Another collection of 39 urethritis-associated *N. meningitidis* identified 4 *cnl* isolates and 17 MenNG isolates due to various mutations in the *cps* locus [91]. More recently, a meningococcal clade of cc11.2 lineage (US_NmUC) with a nongroupable phenotype due to deletion of capsule biosynthesis genes has caused unprecedented clusters of meningococcal urethritis in heterosexual men [6,8,92]. As one example, in Columbus, Ohio from 2015 to 2016, ~25% of presumed gonococcal urethral infections were determined to be meningococcal urethritis with clinical presentation mirroring that of gonococcal urethritis [8,92]. Other mucosal infections, e.g., neonatal conjunctivitis, [93] and at least five cases of IMD (meningitis and meningococcemia) were also reported with this clade [6], although it is not known if these patients were immunocompetent.

While urogenital colonization and sporadic cases of urethritis caused by *N. meningitidis* are documented across many genotypes and groups [90,91,94], the US_NmUC is unique in its capability of causing multicity epidemiologically unlinked urethritis clusters and US_NmUC appears to be sexually transmitted, like gonococci [8,92]. These observations suggest that a phylogenetically distinct nongroupable cc11 US_NmUC has emerged as a new urotropic pathotype to cause meningococcal urethritis. Specific signatures universal to the US_NmUC include (1) an IS1301-mediated specific deletion of the group C capsule biosynthesis genes, (2) expression of a unique FHbp ID896 protein [95], and (3) the acquisition of gonococcal NorB-AniA denitrification apparatus [7]. These unique features differ from other urogenital meningococcal isolates, many of which express capsule, encode a frame-shifted *fHbp* allele, and have a meningococcal denitrification pathway [91,94]. Loss of capsule has been demonstrated to enhance colonization at the mucosal surface, confer increased invasion into epithelial cells [96], and facilitate biofilm formation [97,98]. The acquisition of gonococcal denitrification pathway likely contributes to the success of this clade in better adapting to the male urethra.

Enhanced national surveillance and whole genome sequencing analysis of invasive, urogenital and rectal isolates at CDC has identified ~300 isolates from over 13 states to be members of US_NmUC [6]. This is certainly an underestimate due to possible misidentification by *N. gonorrhoeae* diagnostic assay [99]. In 2019, two US_NmUC isolates were reported in MSM in the United Kingdom [100]. One of the UK isolates had acquired a frameshifted gonococcal maltose phosphorylase gene, resulting in a carbohydrate utilization profile more typically associated with gonococci [100]. Ecological separation within the human host is proposed as an explanation for the lower frequency of interspecies recombination noted between naturally competent *N. meningitidis* and *N. gonorrhoeae* [101]. However, among the members of the US_NmUC, the genome content of total length of DNA sequences inferred to have originated from *N. gonorrhoeae* varied substantially from ~5 to ~30 kb [6]. Further, one 2015 isolate had a gonococcal-like *mtrR* allele that is associated with elevated azithromycin MICs [6] and 7 out of 10 isolates recovered during 2018–2019 in St. Louis, MO, are non-susceptible to azithromycin [99]. In addition, intermediate penicillin resistance was seen in the clade, and one UK isolate, having acquired part of a gonococcal DNA gyrase (*gyrA*) gene, was resistant to ciprofloxacin [100]. These multiple recombination events demonstrated widespread acquisition of gonococcal DNA by US_NmUC and suggested that co-colonization of these two species had facilitated genetic exchanges and raised the prospect of further acquisition of gonococcal antibiotic resistance determinants [100].

## 3. Population Structure of Invasive “Minor” Capsular Groups and Nongroupable *N. meningitidis*

**Figure 1:**
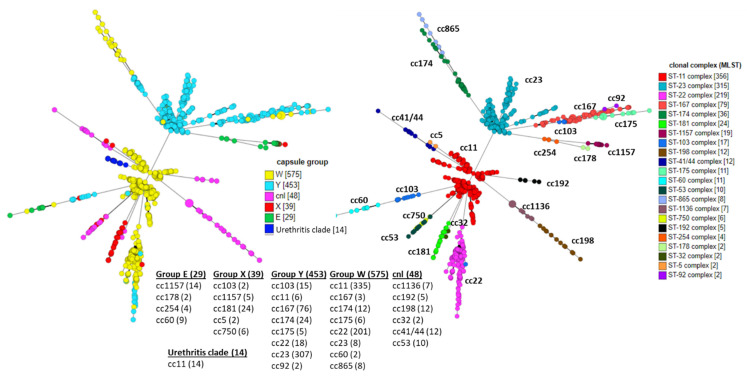
Meningococcal invasive isolates of capsule groups E, W, X, Y, nongroupable *cnl,* and urethritis clade isolates that have whole genome sequencing data and “disease” record entries of “invasive,” “meningitis,” “septicemia,” or “meningitis and septicemia” were retrieved from PubMLST database (http://pubmlst.org/neisseria/, access on 2 March 2021). Isolates without records of “year” and “clonal complex” were excluded. A single isolate that has a unique combination of country/year/sequence type (ST) definition was selected and the clonal complexes with at least two isolates were included in the analysis. The minimum spanning trees were generated and visualized with GrapeTree [103], a plug-in analysis tool at PubMLST, with the scheme of *N. meningitidis* core genome MLST (cgMLST) v 1.0 and default parameters. The trees are colored by capsular groups (**left**) or clonal complexes (**right**). The clonal complex breakdowns of each capsule groups are also listed.

The genetic relationships of W, X, Y, E, and NG capsular groups associated with disease are shown in Figure 1. Genome allelic profile comparisons were made based on the core genome MLST (cgMLST v1.0) scheme with a set of 1605 loci present in ≥95% *N. meningitidis* isolates [102]. The analysis examines 1158 disease-causing isolates: 29 MenE, 575 MenW, 39 MenX, 453 MenY, and 62 MenNG (48 *cnl* and 14 urethritis clade) isolates, compiled by selecting a representative isolate that had a unique country/year/ST combination. Isolates not assigned to a clonal complex or without records of year and country origin were excluded. The resultant minimum spanning tree was visualized by GrapeTree [103], which is integrated into the BIGSdb functionality [104]. As shown, the geographically and temporally diverse collection of disease-causing X, E, *cnl,* and urethritis isolates displayed two distinct major groupings that are dominated by the W:cc11 and Y:cc23 isolates. However, the emergence of multiple distinct clonal lineages—cc11, cc22, cc174, and cc865 in MenW; cc181 and cc750 in MenX; cc60 and cc1157 for MenE; and the distinct cc11 urethritis clade—is evident.

## 4. Meningococcal Vaccines

Capsular polysaccharides have historically been the targets for group-specific meningococcal vaccine development. The first capsular polysaccharide vaccines were developed in the early 1970s. While a major step forward, they were generally not effective for children less than 2 years and failed to induce long-term memory responses. The subsequent development and introduction of polysaccharide–protein conjugate vaccines in the late 1990s markedly accelerated the prevention of meningococcal disease. Meningococcal polysaccharide–protein conjugate vaccines against groups A, C, Y, and W, developed as monovalent, bivalent, or quadrivalent products, have considerably greater effectiveness than the polysaccharide vaccines and induce immune memory responses [105]. The polysaccharide conjugate vaccines reduce transmission by prevention of meningococcal acquisition, resulting in significant herd or community protection at quite modest levels of vaccine coverage. The introduction of a monovalent MenC conjugate vaccine in 2000 virtually eliminated the incidence of MenC disease in United Kingdom, an effect that has persisted for well over two decades, demonstrating 90% effectiveness at 3 years in 11–18-year-olds [106]. Vaccination against MenC induced herd protection and reduced the rates of MenC carriage and disease in non-vaccinated individuals by more than 50% [107]. A MenACWY conjugate vaccines was first licensed in the United States in early 2005. Additional quadrivalent MenACWY conjugate vaccines were subsequently introduced and are now in use globally (Table 1). The group B capsule has not been developed as a vaccine target given its structural similarity with human polysaccharide antigens. However, two outer membrane protein-based vaccines targeting MenB (Table 1), also with activity against non-MenB *N. meningitidis,* have been licensed and are now in use in meningococcal disease prevention strategies.

However, meningococcal vaccination strategies with limited capsular group coverage will eventually select for or uncover previously “minor” capsular groups, now causing significant endemic and epidemic meningococcal disease in the last two decades (e.g., groups W, X, and Y). Examples are the Hajj MenW outbreaks and the emergence of MenW disease in South America and Europe. In addition, the dramatic control of MenA disease in the African meningitis belt achieved by the introduction of MenAfriVac in 2010 uncovered outbreaks of MenX and MenW [39]. Due to horizontal gene transfer and recombination *N. meningitidis,* like *Streptococcus pneumoniae,* can undergo capsule structural change, e.g., “capsule switching” [55,108,109,110,111] lessening herd immunity. Transformation and homologous recombination of capsule genes with the appearance of otherwise identical MenC strains was first noted during a prolonged MenB outbreak in the 1990s [108]. The MenW outbreaks associated with the Hajj in 2000 may have been the result of a historic capsule switching event from cc11 MenC strains. In large meningococcal isolate collections, capsule switching is detected in ~3% of isolates [112].

Pentavalent meningococcal conjugate vaccines, i.e., MenABCWY (MenACWY-Oligosaccharide diphtheria CRM_197_ conjugate, combined with MenB multicomponent recombinant, GlaxoSmithKline), MenABCWY (bivalent FHbp-containing pentavalent vaccine, Pfizer), and MenACXWY (NmCV-5, Serum Institute of India), are in phase 3 clinical trials and are a next step, if widely implemented, for global control of meningococcal disease. The broad capsule focused coverage together with the MenB protein component(s) can potentially provide protection [113] against other minor disease-causing groups and nongroupable strains [95,114,115].

## 5. Conclusions

**Figure 2:**
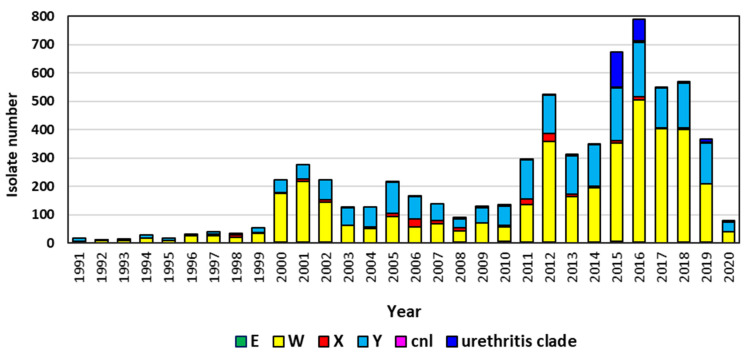
*N. meningitidis* capsular groups E, W, X, Y, and cnl from invasive meningococcal disease and urotropic meningococci submitted to PubMLST database, 1991–2020.

Capsular groups W, X, and Y now cause significant IMD as reflected in the higher numbers of invasive isolates deposited into PubMLST since 2000 (Figure 2) as well as the country and global surveillance data noted above. In addition, group E and nongroupable meningococci have appeared as a cause of invasive disease, and a nongroupable *N. meningitidis* pathotype of the hypervirulent cc11 is causing sexually transmitted urethritis cases and outbreaks. *N. meningitidis* is a human microbe circulating within populations. Due to factors including the introduction of highly effective meningococcal vaccines of limited coverage, the capsular groups causing IMD has changed over time and across geographic regions. Pentavalent meningococcal conjugate vaccines in phase 3 clinical trials appear to be an important next step for enhanced global control. However, the capacity of meningococci to continue to evolve is significant. Genetic transformation and recombination, including transfer of genes between meningococci, gonococci, and commensal *Neisseria* spp. [6,116] and immune selection can all result in the rise, diversification, and disappearance of virulent meningococcal clones. Continued surveillance including molecular characterization is key to recognizing the changing epidemiology of meningococcal disease.

## Figures and Tables

**Table 1:** Meningococcal protein andpolysaccharide conjugate vaccines *.

Vaccine Product	Trade Name	Age Group	Year Licensed
**Polysaccharide Conjugate (Groups A, C, W, and Y)**			
MenACWY-D	Menactra	9 months–55 years	2005
MenACWY-CRM	Menveo	≥2 months	2010
MenACWY-TT	MenQuadfi ^#^/Nimenrix	≥1 year/≥6 weeks	2020/2012
MenA-TT	MenAfriVac	3 months–29 years	2010
**Protein based (directed at group B)**			
MenB-FHbp	Trumenba	10–25 years	2014
MenB-4C ^#^	Bexsero	≥2 months	2015

Abbreviations: MenACWY-CRM = meningococcal groups A, C, W, and Y capsular polysaccharide-diphtheria CRM_197_ conjugate; -D = diphtheria toxoid conjugate; -TT = tetanus toxoid conjugate vaccine; MenB-4C = four-component meningococcal group B vaccine; MenB-FHbp = meningococcal group B bivalent factor H binding protein vaccine. ^#^ MenQuadfi is indicated for ≥2 years in the U.S. Bexsero is licensed for 10–25 years in the U.S. * Monovalent C conjugate vaccines, including Meningitec (MenC-CRM_197_), Menjugate (MenC-CRM_197_), NeisVac-C (MenC-TT), and Menitroix (MenC-TT+Hib), are still in use in some countries. Additional conjugate vaccines directed against MenAC and MenC are also available in China. Pentavalent meningococcal conjugate vaccines: MenABCWY (MenACWY-CRM-197 combined with MenB multicomponent recombinant proteins, GlaxoSmithKline), MenABCWY (bivalent FHbp-containing pentavalent vaccine, Pfizer), and MenACXWY (NmCV-5, Serum Institute of India) are in phase 3 clinical trials.

## Data Availability

Not applicable.
